# Claudin-18.2 mediated interaction of gastric Cancer cells and Cancer-associated fibroblasts drives tumor progression

**DOI:** 10.1186/s12964-023-01406-8

**Published:** 2024-01-10

**Authors:** Shengde Liu, Zizhen Zhang, Lei Jiang, Miao Zhang, Cheng Zhang, Lin Shen

**Affiliations:** Key laboratory of Carcinogenesis and Translational Research (Ministry of Education/Beijing), Department of Gastrointestinal Oncology, Peking University Cancer Hospital & Institute, Beijing, 100142 China

**Keywords:** Gastric cancer, CLDN18.2, cancer-associated fibroblasts, Metastasis, Adhesion, S100A4

## Abstract

**Background:**

Claudin-18.2 (CLDN18.2) has emerged as an alluring therapeutic target against gastrointestinal tumors in recent years. However, a thorough understanding of its regulatory mechanism in gastric cancer remains elusive.

**Methods:**

We presented a comprehensive study comprising 185 gastric cancer patients, which included 112 cases with high CLDN18.2 expression and 73 cases with low CLDN18.2 expression as determined by immunohistochemistry. After overdressed CLDN18.2 in AGS and NUGC4 cell lines, we elucidated the functions of CLDN18.2 in connecting gastric cancer cells and cancer-associated fibroblasts (CAFs) through an in vitro adhesion models and in vivo lung colonization models. The molecular mechanism underlying CLDN18.2-mediated interaction between gastric cancer cells and CAFs was identified through RNA sequencing and protein-proximity labeling techniques in vivo.

**Results:**

In our own cohort, a correlation was observed between high levels of CLDN18.2 expression and advanced cancer stage, poor prognosis, and heightened infiltration of CAFs. We elucidated a pivotal role of CLDN18.2 in mediating adhesion between gastric cancer cells and CAFs, which leads to the adhesion of cancer cells to stroma tissue and facilitates the clustering of cancer cells and CAFs into embolus, enhancing gastric cancer’s metastatic progression and the risk of embolic death. Mechanistically, it was discovered that CAFs can activate adhesion and metastasis-related signaling pathways in CLDN18.2-positive gastric cancer cells. Furthermore, using an in vivo protein-proximity labeling approach, we identified S100 calcium binding protein A4 (S100A4) as a distinctive marker of CAFs that interacts with CLDN18.2 to enhance gastric cancer progression.

**Conclusions:**

Our findings illuminated the role of the CLDN18.2-mediated interaction between cancer cells and CAFs in promoting gastric cancer progression and embolism, thereby providing insight into potential therapeutic avenues for CLDN18.2 positive cancers.

Video Abstract

**Supplementary Information:**

The online version contains supplementary material available at 10.1186/s12964-023-01406-8.

## Introduction

Gastric cancer (GC) ranks as the fifth most frequently occurring cancer globally and is associated with high incidence and mortality rates [1]. Despite significant progress in diagnostic and treatment modalities, the prognosis of GC patients remains poor, emphasizing the pressing need to identify new therapeutic targets and effective drugs to improve patient outcomes [2]. Combining such treatments with personalized medicine techniques may provide a promising approach towards achieving this goal.

Claudin-18.2 (CLDN18.2) is a compelling target for GC therapy due to its selective expression in cancer cells and significant role in tumor progression [3, 4]. Preclinical studies have shown that monoclonal antibodies or CAR-T cells targeting CLDN18.2 could inhibit tumor growth, increase the survival rates of GC patients [5]. These promising preclinical findings have motivated the development of clinical trials [6–10]. However, not all GC patients have positively responded to CLDN18.2-targeted therapy [9, 10]. Several clinical studies have reported primary resistance to anti-CLDN18.2 therapy. Although the underlying mechanisms of resistance to CLDN18.2-targeted therapy are not entirely clear, several potential factors have been proposed. Firstly, studies have shown that CLDN18.2 expression is heterogeneous among GC patients, with some tumors not expressing CLDN18.2 at all [11]. Secondly, the loss of CLDN18.2 expression during disease progression may contribute to acquired resistance [12]. Thirdly, the presence of TME components, such as CAFs, may contribute to therapy resistance. Therefore, the identification of potential resistance mechanisms and developing strategies to overcome these obstacles will be crucial for the success of CLDN18.2-targeted therapy.

Cancer-associated fibroblasts (CAFs) are a crucial component of the tumor microenvironment, with diverse functions in tumor initiation, progression, invasion, and metastasis that have been extensively studied [13–15]. Recent research has specifically highlighted their role in promoting gastric cancer progression, by secreting growth factors and cytokines that stimulate tumor cell proliferation and angiogenesis [16]. In addition, CAFs contribute to the invasiveness of cancer cells by facilitating extracellular matrix remodeling and promoting the epithelial-mesenchymal transition (EMT) [17]. The immune response against cancer is also influenced by CAFs, as they can suppress T cell function and promote the recruitment of immunosuppressive cells such as regulatory T cells and myeloid-derived suppressor cells, thus contributing to immune evasion [18]. Furthermore, the characterization of CAFs heterogeneity in gastric cancer has revealed their diverse and distinct subtypes, such as myofibroblastic CAFs, inflammatory CAFs, and antigen-presenting CAFs, with different functions in regulating gastric cancer progression [19]. Further investigation into the complex interactions between CAFs and tumor cells is needed to develop effective strategies for the treatment of gastric cancer. A thorough understanding of these interactions will assist in the development of CAFs-targeting therapies aimed at disrupting their contribution to cancer progression.

In this study, we verified that CLDN18.2 can mediated the interaction between gastric cancer cells and CAFs to promote the progression of gastric cancer. According to our own gastric cancer cohort, CLDN18.2-positive patients had higher stage, poorer prognosis, and more CAFs infiltrated into tumors than CLDN18.2-negative patients. Based on phenotypic, omics and molecular evidences, we proved that a characteristic marker of CAFs, S100A4, interacts with CLDN18.2 to accelerate the metastatic progression of gastric cancer in a CAF-dependent manner. Our work provided new insights into the mechanism of action of CLDN18.2 in regulating the progression of gastric cancer, and proposed combining CAFs inhibition as an improvement for current CLDN18.2-targeted therapies.

## Materials and methods

### Clinical samples

A total of 185 patients with gastric cancer (GC) were enrolled in the study at Peking University Cancer Hospital. All patients’ tissue samples were preserved using formalin-fixed, paraffin-embedded (FFPE) methods. Pathologists classified all tissue samples as adenocarcinomas, and calculated H-scores for CLDN18.2 staining. Prior to conducting experiments with patient samples, patient consent was obtained and the Ethics Committee of Peking University Cancer Hospital approved all studies involving clinical samples.

### Cell lines

AGS and NUGC4 cell lines were obtained from the Chinese Academy of Medical Sciences (Beijing, China), and HEK293T (Human Embryonic Kidney 293 T) cells were obtained from ATCC. HEK293T cells were cultured in DMEM medium (Gibco) supplemented with 10% fetal bovine serum (FBS) (Gibco) and 100 U/ml Penicillin-Streptomycin at 37 °C with 5% CO_2_. Additionally, all cancer cells were maintained in 1640 medium supplemented with 10% FBS and 100 U/ml penicillin/streptomycin.

### Antibodies and plasmid

Anti-Claudin18.2 (ER1902–86), anti-FAP (ET1704–23), anti-S100A4 (ET1612–13), anti-GAPDH (ET1601–4), anti-β-actin (R1207–1), and anti-DYKDDDDK Tag (FLAG) (0912–1) were obtained from Hangzhou HuaAn Biotechnology. The anti-α-SMA (14395–1-AP), HRP-conjugated Streptavidin (SA00001–0), and FlexAble CoraLite® Plus 555 antibody (KFA002) labeling kit were provided by Proteintech. The anti-Claudin18.2 inhibitory antibody, Zolbetuximab (HY-P99058), was supplied by MedChemExpress. In all experiments, plasmids were constructed using Gibson assembly cloning techniques as previously [20]. Full-length human CLDN18.2 was amplified from cDNA of AGS cells with the forward primer: 5′- ATGGCCGTGACTGCCTGTCA-3′ and reverse primer: 5′- CACATAGTCGTGCTTGGAAGGATA − 3′.

### Western blot

Cells or tissues were washed with PBS and lysed with RIPA lysis buffer on ice for 30 minutes. After separation by SDS/PAGE, proteins were transferred onto 0.45 μM polyvinylidene difluoride (PVDF) membranes and incubated overnight with primary antibodies. This was followed by incubation with HRP-conjugated secondary antibodies and detection by enhanced chemiluminescence.

### RNA extraction and quantitative real-time PCR (RT-qPCR)

Total RNA was extracted using the TRNzol Universal Reagent (TIANGEN, DP424) according to the manufacturer’s protocol. The further reverse transcription reaction was carried out using the PrimeScript™ RT reagent Kit (Takara, RR037Q). Quantitative real-time PCR was performed using the Premix Ex Taq™ (Probe qPCR) (Takara, RR39LR). All experiments were independently performed in triplicate. The results are expressed as relative gene expression levels normalized to *GAPDH*. The primer sequences of the human genes related to this study are listed in Table. S3. For targeted gene knockdown, siRNA was procured from GenePharma (Suzhou, China). The specific siRNA sequences utilized in this study are detailed in Table. S4.

### Isolation and culture of CAFs

In this study, fresh human gastric cancer tissues and adjacent normal tissues were obtained from Peking University Cancer Hospital for this study. Before uniformly pressing the GC tissues onto the culture plate, they were dissected into small fragments with 2 mm diameters. CAFs were allowed to migrate in a 37 °C, 5% CO_2_ incubator with 20% FBS-DMEM medium for 1–2 weeks, after which they were purified by enzyme digestion. Finally, CAFs were verified by western blotting with FAP, S100A4, and α-SMA.

### Cancer cell-fibroblast adhesion assay

To form a confluent monolayer, 6 × 10^4^ CAFs were seeded per well in 500 mL of DMEM medium in a 24-well plate. GC cells were either labeled with GFP or stained with CellTrac CFSE, and their numbers were counted using the Countess 3 Automated Cell Counter. The medium containing the fibroblasts was then removed and replaced with freshly suspended RPMI 1640 medium containing 5 × 10^4^ GC cells. To allow GC cells to adhere to the fibroblasts, the plate was incubated at 37 °C for 0.5 hours. Non-adherent GC cells were gently washed away with PBS, and the attached GC cells were counted under a fluorescence microscope.

### Cancer cell-fibroblast cocultured migration and invasion assay

For migration and invasion assay in the co-culture models, 10 × 10^4^ GC cells were either labeled with GFP or stained with CellTrac CFSE. GC cells and CAFs were resuspended in RPMI 1640 medium containing 1% FBS at a 10:1 ratio and seeded into each transwell. Following a 24-hour incubation to remove non-migrating cells from the upper chamber, migrated GC cells were photographed and counted under a fluorescence microscope. In this experiment, regardless of whether cultured in RPMI 1640 or DMEM, no significant differences in growth rate or cellular state were observed among CAFs.

### Lung colonization experiment in vivo

All animal experiments were performed under the guidelines of the Institutional Animal Care and Use Committee of the Beijing Cancer Hospital (Ethics Approval Number: EAEC 2022–04). AGC cells labeled with GFP were injected into nude mice via their tail veins, with or without co-injection with CAFs (5 × 10^6^ AGS-GFP cells and 5 × 10^5^ CAFs per mouse) [21]. After 24 hours of inoculation, the pulmonary blood vessels were perfused with PBS, and the colonized AGC-GFP cells were analyzed by immunofluorescence.

### Immunohistochemistry and paraffin immunofluorescence

Paraffin-embedded samples were baked, deparaffinized, and rehydrated before antigen retrieval and nonspecific antibody staining. Sections were then incubated with primary antibodies against CLDN18.2 followed by secondary antibodies. CLDN18.2 staining was independently estimated by two pathologists using a light microscope. Positive staining cells and the intensity of GC samples were scored and evaluated. A final H-score ranging from 0 to 270 was calculated based on the percentage of positive tumor cells [22]. For paraffin immunofluorescence assays, sections were first immobilized with 4% paraformaldehyde, and then blocked with 5% bovine serum albumin after antigen retrieval by immunohistochemistry. Afterwards, sections were incubated with primary and secondary antibodies.

### Proximity-dependent biotin identification in vivo

The lentivirus was used to construct SP-TurboID-PDGFRB-TM or TurboID-CLDN18.2 stable expressed AGS cells. Then, a nude mouse model of AGS subcutaneous xenografts was developed. Two weeks later, mice received 500uM biotin intraperitoneally for 60 minutes before harvesting tumor cells as previously described [23]. Tumor cells were lysed in RIPA lysis buffer at 4 °C for 10 minutes. After washing twice with RIPA lysis buffer, streptavidin magnetic beads were mixed with the supernatant and rotated overnight at 4 °C. The following day, the beads were washed three times with RIPA lysis buffer. For mass spectrometry, biotinylated proteins were eluted by boiling them in 150 ml of elution buffer (55 mM pH 8.0 Tris-HCl, 0.1% SDS, 5 mM DTT, and 0.5 M biotin).

### RNA sequencing

Total RNA was extracted using the TRIzol reagent (Invitrogen) and libraries for sequencing were generated using the NEB Next Ultra Directional RNA Library Prep Kit for Illumina (NEB), following the manufacturer’s recommendations. The quality of the library was assessed using an Agilent 2100 Bioanalyzer with respect to product size and concentration. The paired-end libraries were sequenced using the Illumina HiSeq 2500 platform, with a read length of 150 bp. FASTP (v2.2.0) was utilized to eliminate adapters and low-quality reads. Alignment of paired-end reads to the human genome (hg19) was executed with HISAT2 (v2.1.0). The expression levels of each gene in each sample were determined by StringTie (v1.3.4) as FPKMs. Differentially expressed genes were identified utilizing DEseq2, using a *P* value < 0.05 and |log2 (fold change)| > 1 as significant cut-offs.

### Statistical analysis

All bar graphs were analyzed using GraphPad Prism version 8. The data presented in this study represent the mean ± SEM from triplicate experiments. Two-tailed Student’s t-tests were used for all comparisons between two variables. The Kaplan-Meier curves were compared using the log-rank test, and Spearman correlation analysis was used to assess correlations between two variables. Statistical significance was designated as ****P < 0.0001, ***P < 0.001, **P < 0.01, *P < 0.05, or ns (*P* > 0.05).

## Result

### CLDN18.2 expression correlates with clinical staging in gastric Cancer

**Fig. 1:**
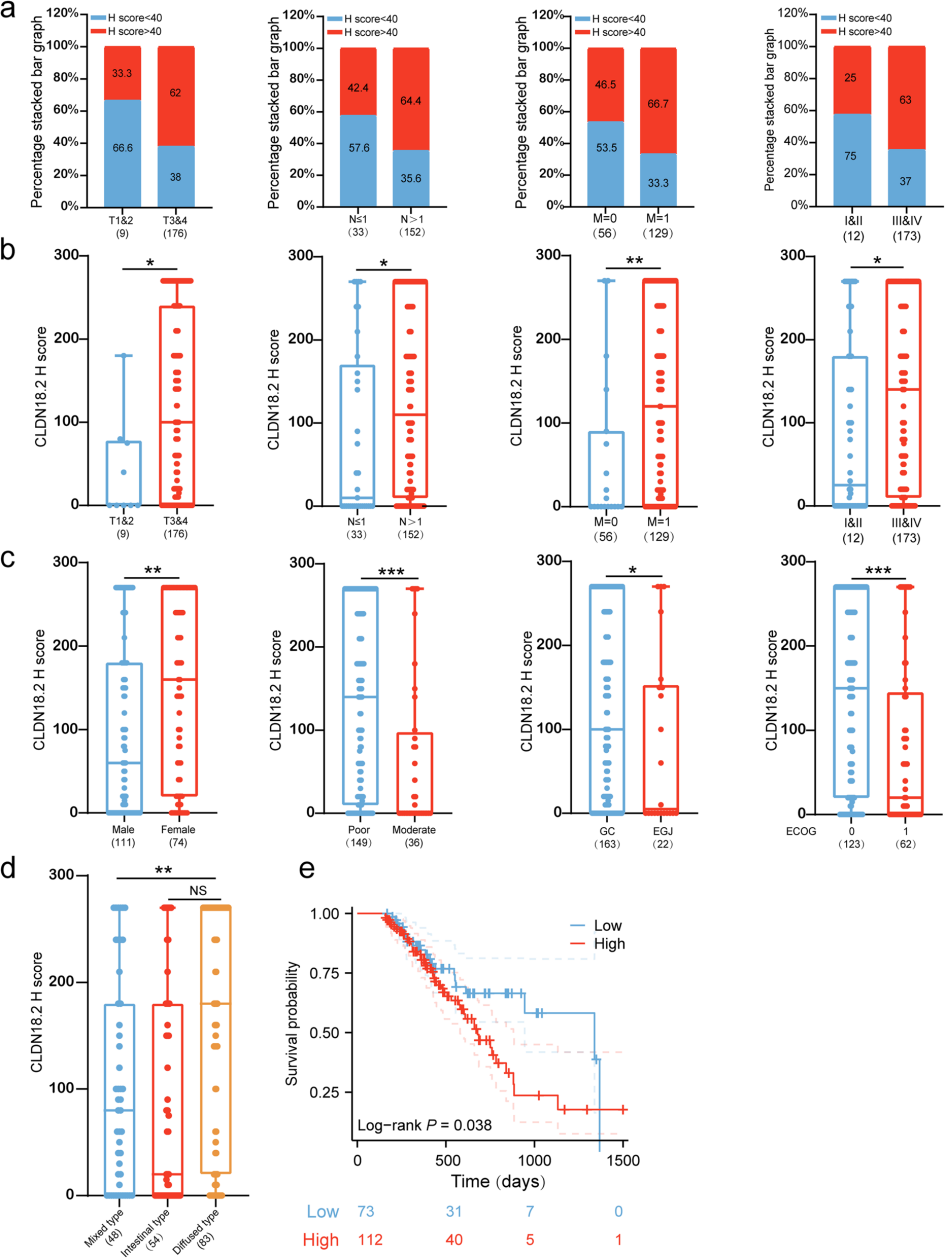
CLDN18.2 Expression Correlates with Clinical Staging in Gastric Cancer. **a**. The chi-square test was used to compare the CLDN18.2 H-score > 40 and H-score < 40 in different TNM stages in our cohort. **b**. The comparison of CLDN18.2 H-score in different TNM stages in our cohort, student t-test was utilized. **c**-**d**. The comparison of CLDN18.2 H-score in different clinicopathologic features in our cohort was performed. **e**. The overall survival of patients was based on CLDN18.2 expression in our cohort, which was evaluated using the log-rank test. The statistical significance was denoted as * *P* < 0.05, ** *P* < 0.01, *** *P* < 0.001, and not significant (ns)

In this study, we aimed to investigate the expression characteristics of CLDN18.2 in gastric cancer clinical staging and its correlation with other pathological molecular characteristics. We analyzed 185 gastric cancer patients enrolled in our department by performing immunohistochemical staining and evaluating the H-score. Our results revealed that patients with low CLDN18.2 expression (H-score < 40; *n* = 73) were more likely to have lower TNM stages, whereas patients with high CLDN18.2 expression (H-score > 40; *n* = 112) were more likely to have higher TNM stages (*P* < 0.01) (Fig. 1a-b; Table. S1). Moreover, we found that poorly differentiation, diffuse Lauren subtype, and primary location were associated with a higher expression level of CLDN18.2 in gastric cancer tissue, whereas CLDN18.2 expression was lower in patients with an Eastern Cooperative Oncology Group (ECOG) score of ≥1 and male patients (Fig. 1c-d). Furthermore, we analyzed the prognostic value of CLDN18.2 in our cohort and observed that high expression of CLDN18.2 (H-score > 40) was significantly linked to poorer overall survival (OS) (*P* < 0.05) (Fig. 1e). Taken together, our findings suggest a positive correlation between CLDN18.2 expression in gastric cancer tissues and clinical stage, as well as poor prognosis of gastric cancer.

### CLDN18.2 expression shows a positive correlation with the level of CAFs infiltration

**Fig. 2:**
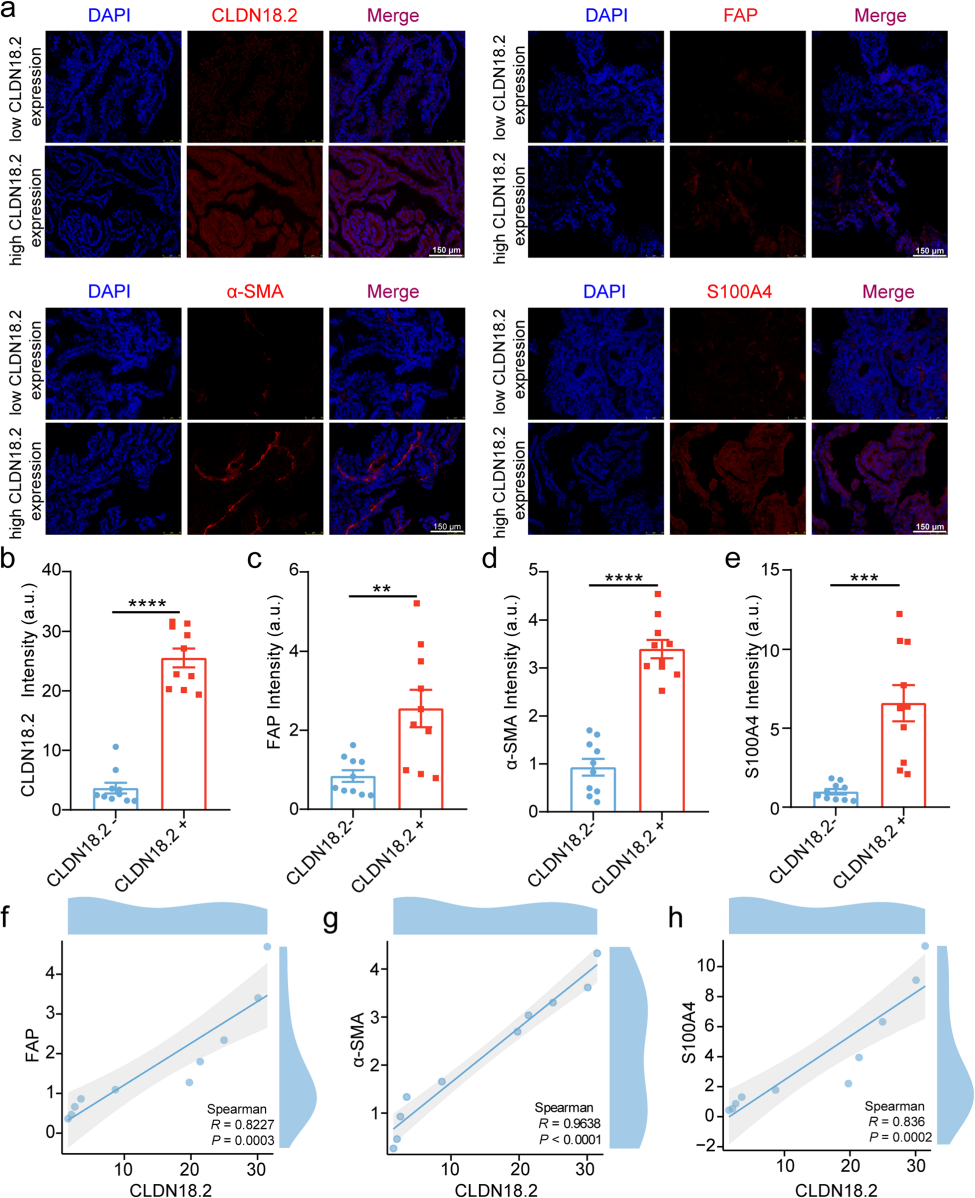
CLDN18.2 expression shows a positive correlation with the level of infiltration by CAFs. **a**. PDX tumors with negative and positive CLDN18.2 staining was analyzed using paraffin immunofluorescence with anti-CLDN18.2 (red), anti-FAP (red), anti-α-SMA (red), and anti-S100A4 (red) antibodies. Nuclei were counterstained with DAPI (blue). Scale bars represent 150 μm. **b**-**e**. The quantitative analysis of immunofluorescence intensity of CLDN18.2, FAP, α-SMA, and S100A4 expression was performed based on the results shown in Fig. 2a. **f**-**h**. The Spearman correlation analysis was used to assess the relative expression levels of FAP, α-SMA, S100A4, and CLDN18.2, as shown in Fig. 2b-e. all data were shown as the mean ± SEM, and data were analyzed using Student’s t-tests, **P < 0.01, ***P < 0.001, and ****P < 0.0001

CLDN18.2 is a tight junction protein that interacts with different cell types in the tumor microenvironment, such as epithelial cells and CAFs [24]. Among these cells, CAFs have been shown to have a crucial role in the progression and metastasis of gastric cancer [25]. To investigate the underlying mechanism of CLDN18.2 in the promotion of gastric cancer, we evaluated the infiltration of CAFs in PDX tissue sections using IHC (Fig. 2a; Table. S2). Our analysis in the xenograft tissue sections of gastric cancer PDX models revealed a significant increase in the expression of CAFs markers FAP, α-SMA, S100A4 in the high CLDN18.2 expression group compared to the low CLDN18.2 expression group (Fig. 2b-e). These findings suggested a positive correlation between CLDN18.2 and CAFs infiltration in gastric cancer cells within the tumor microenvironment. Further statistical analysis using the Spearman non-parametric test showed a significant positive correlation (*P* < 0.001) between CLDN18.2 expression and above mentioned CAFs-related markers (Fig. 2f-h). Thus, our results demonstrate the positive correlation between CLDN18.2 and CAFs infiltration, highlighting the significance of this interaction in the progression of gastric cancer.

### CAFs enhance the in vitro adhesion and migration of gastric cancer cells expressing CLDN18.2

**Fig. 3:**
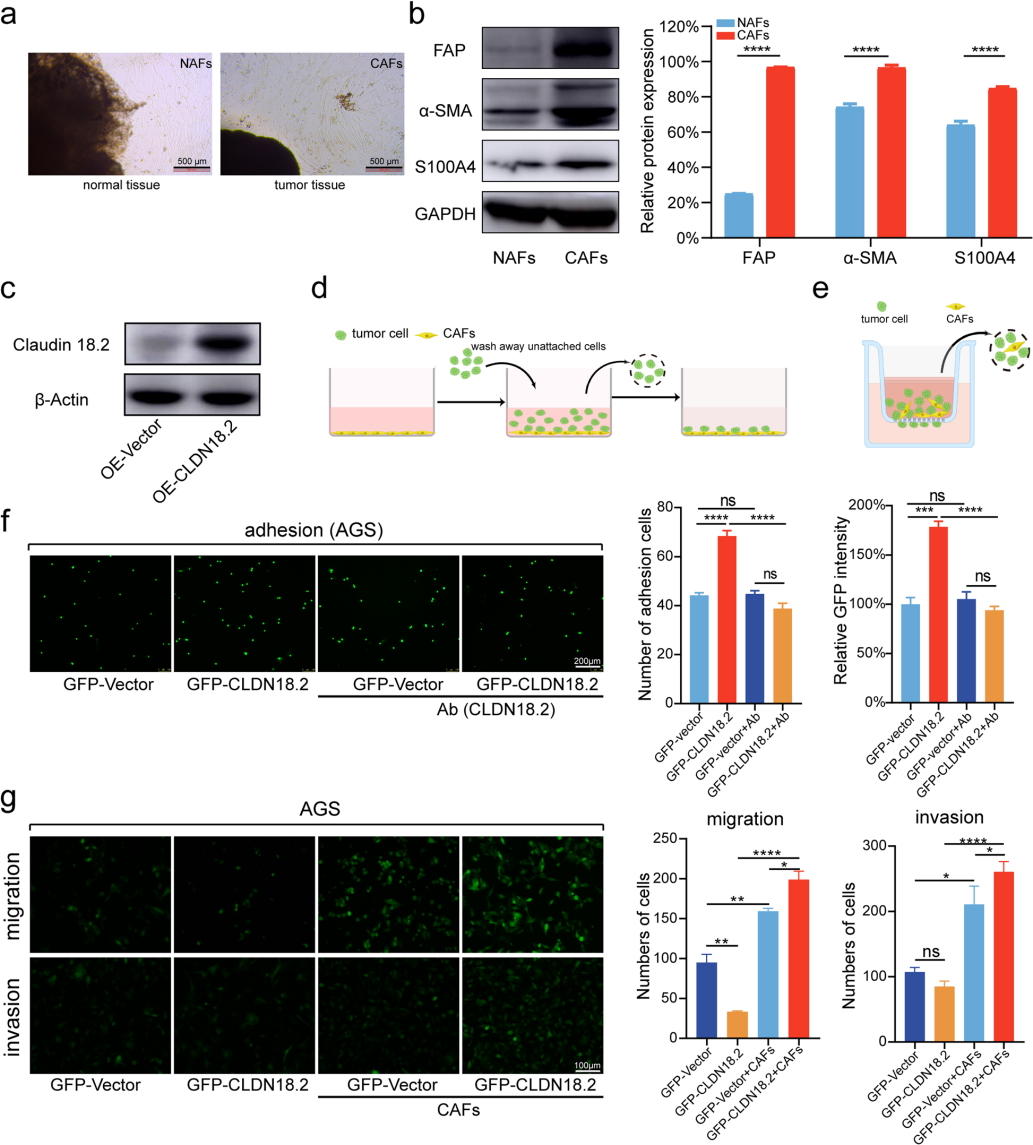
CAFs enhance the adhesion and migration of gastric cancer cells expressing CLDN18.2 in vitro. **a**. Representative images of CAFs were employed to verify the reliability of CAFs isolated from gastric cancer tissue. **b**. The protein levels of FAP, α-SMA, and S100A4 in CAFs were evaluated based on the results shown in Fig. 3a. The data were shown as the mean ± SEM and analyzed using Student’s t-tests, ****P < 0.0001. **c**. The protein levels of CLDN18.2 were measured in AGS cells infected with CLDN18.2 lentivirus. **d**. The schematic diagram depicting the gastric cancer cell-CAFs adhesion model was provided. **e**. The schematic diagram of the gastric cancer cell-CAFs co-culture migration assay was illustrated. **f**. Adhesion of AGS cells expressing GFP or GFP-CLDN18.2 to CAFs was evaluated in the presence or absence of CLDN18.2 antibody blocking, and representative images are shown. The scale bar represents 200 μm. **g**. AGS cells expressing GFP or GFP-CLDN18.2 were co-cultured with or without CAFs, and their migration and invasion abilities were measured. Statistical analysis was performed accordingly, and the scale bar represents 100 μm. The data were shown as the mean ± SEM and analyzed using one-ANOVA test in Fig. 3f, g, *P < 0.05, **P < 0.01, ***P < 0.001 and ****P < 0.0001, ns: no significant

To further explore the potential role of CLDN18.2 in gastric cancer metastasis and progression through CAFs, we extracted primary CAFs from tumor tissues of gastric cancer patients and normal associated fibroblasts (NAFs) from adjacent non-cancerous tissues (Fig. 3a). Using Western blotting and quantitative PCR, we found that the expression levels of FAP, α-SMA, and S100A4 were significantly higher in CAFs compared to NAFs (Fig. 3b), confirming the successful extraction of primary CAFs from gastric cancer patients. Previous studies have suggested that CAFs play a crucial role in tumor progression by interacting with cancer cells. To investigate whether CLDN18.2 mediates the interaction between gastric cancer cells and CAFs, we established an in vitro adhesion model at the cellular level (Fig. 3d). Our results showed that overexpression of CLDN18.2 increased the adhesion of AGS or NUGC4 cells to the underlying layer of CAFs (Fig. 3c, f; Fig. S1a-c). Moreover, we examined the potential of CLDN18.2 antibodies to disrupt the adhesion process between gastric cancer cells and CAF cells. The results from our antibody-blocking experiments unequivocally revealed that the inhibition of CLDN18.2, achieved through the administration of antibodies, effectively impeded the adhesive interaction between gastric cancer cells and CAF cells (Fig. 3f; Fig. S1b-c). Conversely, subsequent investigations focused on the targeted downregulation of CLDN18.2 in SNU-16 cells, leading to a marked reduction in their adhesion to CAFs (Fig. S1d-f). Additionally, the application of siRNA to downregulate CLDN18.2 in AGS cells, known for their stable CLDN18.2 expression, consistently yielded the outcome of reduced adhesion between AGS cells and CAFs (Fig. S1g-i). To further assess the impact of CAFs on the migration and invasion of gastric cancer cells, we developed a co-culture model of gastric cancer cells and CAFs (Fig. 3e). Our results demonstrated that CAFs significantly promoted the migration and invasion of stable CLDN18.2-positive AGS cells compared to the stable GFP AGS group (Fig. 3g). Consistent with our earlier observations, the siRNA-mediated downregulation of CLDN18.2 in AGS cells with stable CLDN18.2 expression effectively impeded the cell migration process (Fig. S1j-k). Taken together, these findings provide compelling evidence that CAFs promote the in vitro adhesion, migration, and invasion of CLDN18.2-positive gastric cancer cells, suggesting that targeting the interaction between CLDN18.2 and CAFs may represent a potential therapeutic approach for gastric cancer.

### CAFs promote the in vivo adhesion of gastric cancer cells expressing CLDN18.2 to lung endothelial cells

**Fig. 4:**
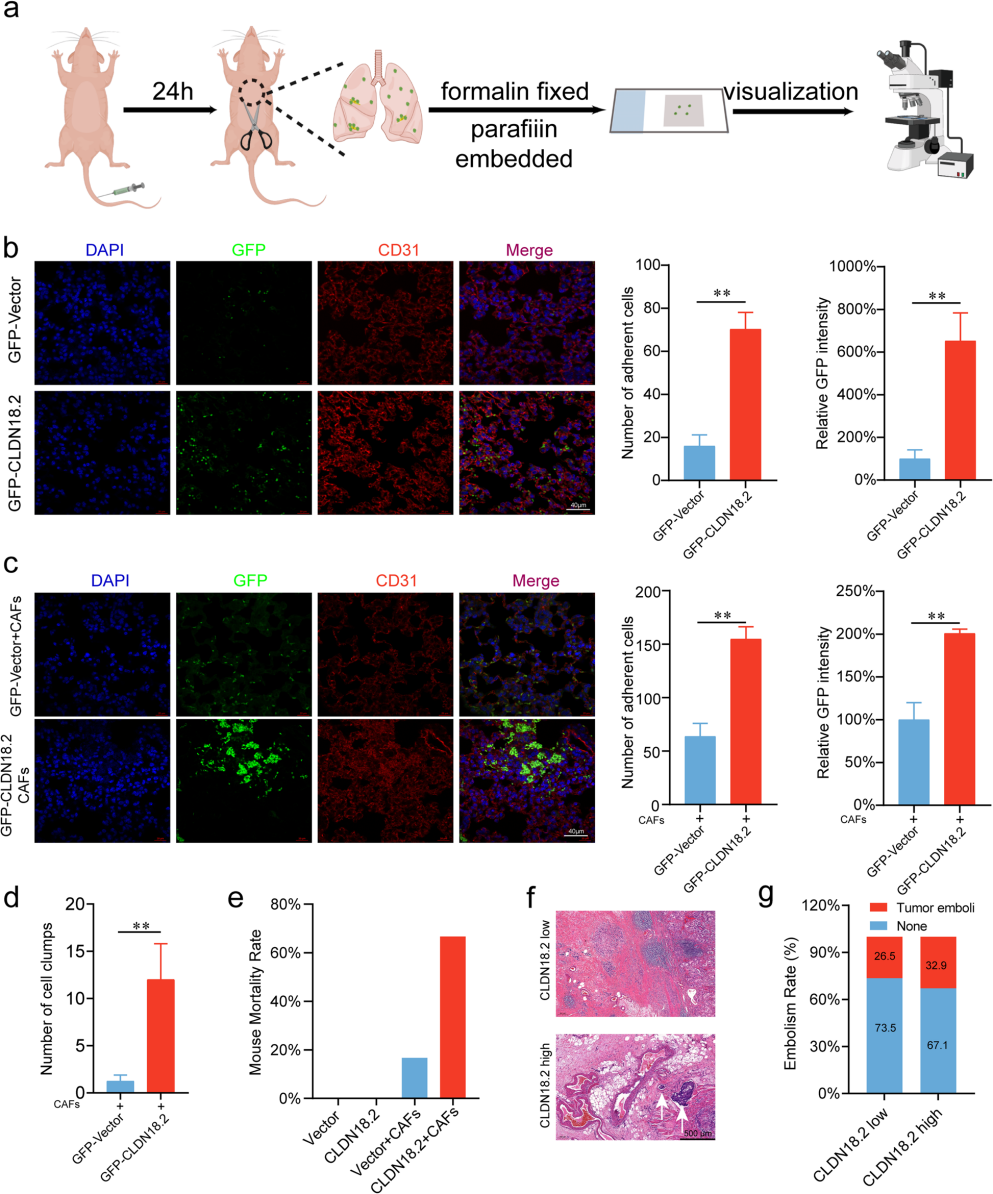
CAFs promote the adhesion of gastric cancer cells expressing CLDN18.2 to endothelial cells in vivo. **a**. Schematic diagram of lung colonization experiment in vivo. **b**. GFP or GFP-CLDN18.2-expressing AGS cells were injected into the mice tail vein, and the colonized cancer cells in the lungs were quantified by paraffin immunofluorescence assay after 24 hours. The blood vessels were stained with CD31 (red), and the nuclei were stained with DAPI (blue). Scale bar represents 40 μm. **c**. Mice were intravenously injected with AGS cells expressing either GFP or GFP-CLDN18.2, in combination with CAFs. The colonized cancer cells in the lungs were quantified by paraffin immunofluorescence assay after 24 hours. The blood vessels were stained with CD31 (red), and the nuclei were stained with DAPI (blue). Scale bar represents 40 μm. **d**. Analysis on the number of cancer cell clusters (> 3 cells) formed in the mouse lung tissue in Fig. 4c. **e**. The mice were injected with either 3 × 10^7^ GFP-Vector or CLDN18.2-GFP AGS cells in combination with or without 3 × 10^6^ CAFs, and the rapid mortality of the mice was assessed within 10 minutes. **f**-**g** Retrospective review of pathology results in GC patients assessed the incidence of tumor embolism in those with high and low CLDN18.2 expression. White arrowheads indicate tumor embolism (**f**). Scale bar represents 500 μm. All data were shown as the mean ± SEM and analyzed using Student’s t-tests, **P < 0.01

CAFs are well known to play critical roles in promoting tumor growth and invasion by altering the tumor microenvironment [26]. To further explore the effects of CAFs on the adhesion and migration of gastric cancer cells in vivo, we constructed an adhesion model in mice (Fig. 4a). We constructed gastric cancer cell lines AGS and NUGC4 stably expressing GFP or CLDN18.2-GFP, then injected these cells into mice through the tail vein, and determined the adhesion of gastric cancer cells in the lung tissue using IHC. Our in vivo results showed that CLDN18.2-positive gastric cancer cells had more adherent numbers on lung endothelial cells compared to GFP-expressing gastric cancer cells. Additionally, GFP-expressing gastric cancer cells co-localized with the lung endothelial cell marker CD31 (Fig. 4b; Fig. S2a). Next, we examined the effect of exogenous CAFs on gastric cancer cell adhesion in vivo. We mixed CAFs with AGS cells or NUGC4 cells stably expressing GFP or CLDN18.2-GFP and injected them into mice through the tail vein. Our results showed that CAFs promoted the adhesion of CLDN18.2-GFP gastric cancer cells to lung endothelial cells, and that tumor cells formed more and larger cell clusters compared with the stable GFP gastric cancer cell group (Fig. 4c-d; Fig. S2b). Meanwhile, we observed that intravenously injecting mice with the same number of CLDN18.2-GFP gastric cancer cells in combination with CAFs significantly increased the incidence of pulmonary embolism and led to a higher mortality rate compared to the control group (Fig. 4e). We hypothesize that the rapid mortality of the mice can be attributed to the formation of cell clusters due to the interaction between CAFs and gastric cancer cells, leading to acute embolism in the mice. The question arises as to whether the coalescence of tumor cells with CAFs precipitates vascular tumor embolism in gastric cancer patients, a phenomenon well-established for its potential to foster distant metastasis. To address this query, we conducted a retrospective examination of clinical case reports in the cohort of gastric cancer patients, endeavoring to quantify the incidence of cancer thrombosis (Fig. 4f-g). Our statistical analysis revealed a higher prevalence of cancer thrombosis in the CLDN18.2 high-expressing group as compared to the CLDN18.2 low-expressing group. Overall, our results provided evidence that CAFs can promote the adhesion of CLDN18.2-positive gastric cancer cells to endothelial cells, which may contribute to the progression of the tumor.

### CAFs stimulation activates adhesion-related signaling pathways in CLDN18.2-expressing gastric cancer cells

**Fig. 5:**
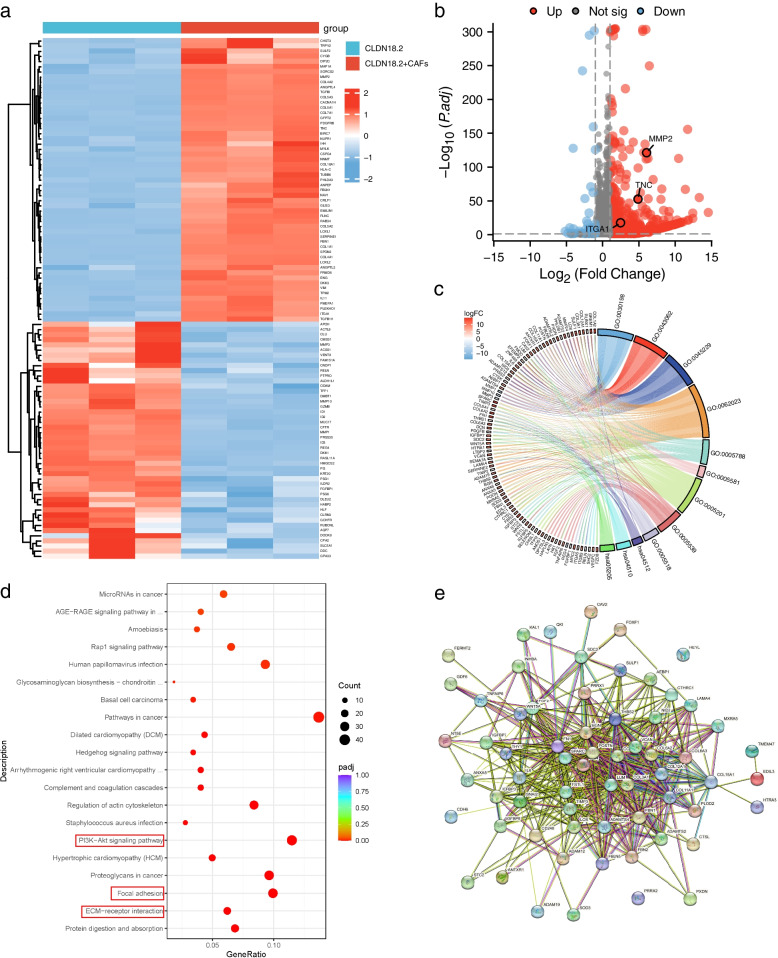
CAFs stimulation induces activation of adhesion-related signaling pathways in CLDN18.2-expressing gastric cancer cells. **a**. RNA sequencing data were generated from CLDN18.2-overexpressing AGS cells co-cultured with or without CAFs. Genes with a *P* value < 0.05 and | log_2_ (fold change) | > 1 were considered significantly differentially expressed. The top 50 upregulated and top 50 downregulated genes were selected for heatmap analysis. **b**. Volcano plot analysis of all up- and downregulated genes was performed. **c**-**d**. GO and KEGG analyses were performed to analyze the differential gene expression. **e**. Protein interaction analysis of differential genes was conducted

To further elucidate the mechanism by which CAFs promote the adhesion and metastasis of gastric cancer cells expressing CLDN18.2, we conducted RNA sequencing on AGS cells co-cultured with CAFs. Our analysis revealed that after co-culturing of CLDN18.2-positive AGS cells with CAFs, the expression of several adhesion-related molecules, including TNC, ITGA1, and MMP2, was upregulated (Fig. 5a-b). Of note is the gene TNC, an extracellular matrix protein renowned for its proclivity to induce angiogenesis and facilitate the EMT process [21]. Our inquiry thus focuses on elucidating whether AGS cells, when co-cultured with CAFs, enhance their adhesion and migration capabilities through the upregulation of TNC. Subsequently, we conducted focused experiments, wherein TNC was knockdown in AGS cells marked by CLDN18.2 overexpression (Fig. S3a). Our ensuing observations during AGS cell and CAFs co-culture have uncovered a significant reduction in adhesion capability within the siRNA-TNC group, as compared to the siRNA-NC counterpart (Fig. S3b-c). These findings underscore the instrumental role played by TNC in driving augmented adhesion between AGS cells and CAFs. Consistently, our efforts to suppress TNC in AGS cells overexpressing CLDN18.2 further substantiated our findings, revealing a concurrent decrease in AGS migration capabilities (Fig. S3d-e). Gene Ontology (GO) enrichment analysis showed significant enrichment in extracellular matrix organization (GO0030198), extracellular structure organization (GO0043062), and collagen fibril organization (GO0030199) biological processes in CLDN18.2 gastric cancer cells co-cultured with CAFs (Fig. 5c). Additionally, Kyoto Encyclopedia of Genes and Genomes (KEGG) enrichment analysis revealed significant enrichment in the ECM-receptor interaction, focal adhesion, PI3K-Akt signaling pathway, and other signaling pathways in CLDN18.2 gastric cancer cells co-cultured with CAFs (Fig. 5d). Differential analysis identified multiple interaction relationships between the genes involved (Fig. 5e). In conclusion, our findings suggested that CAFs promote the activation of adhesion-related pathways in CLDN18.2-positive gastric cancer cells, thus contributing to the metastasis of these cells.

### S100A4 secreted by CAFs promotes the adhesion of CLDN18.2-expressing gastric cancer cells

**Fig. 6:**
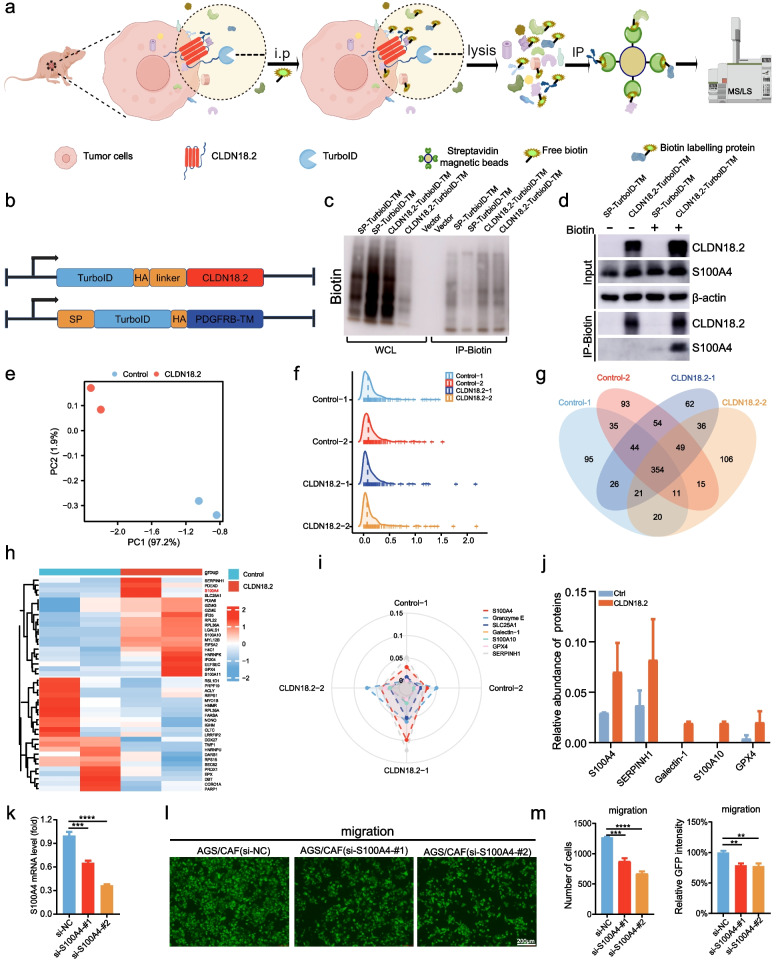
S100A4 secreted by CAFs promotes the adhesion of CLDN18.2-expressing gastric cancer cells. **a**. Schematic diagram of proximity-dependent biotin identification in vivo. **b**. Schematic diagram of plasmid construction. The N-terminus of CLDN18.2 was coupled to HA-turboID with a linker, HA-turboID was coupled to the N-terminus of PDFGRB with a linker. HA: haemagglutinin tag. TurboID: biotin ligase. SP: signal peptide. **c**. Lentivirus was used to stably express SP-TurboID-TM and CLDN18.2-TurboID in AGS cells, which were subsequently employed to form subcutaneous tumors in mice. After intraperitoneal injection of biotin, the tumor tissue from each mouse was subjected to proximity-dependent biotin identification. We present representative photographs of western blots from tumor tissues. **d**. Stable AGS cell lines expressing either SP-TurboID-TM or CLDN18.2-TurboID-TM were utilized for co-culturing with CAFs over 24 hours. Subsequently, 500 μM biotin was introduced to the co-culture system for 60 minutes, facilitating TurboID-mediated biotinylation of proteins proximal to CLDN18.2. Two control groups were maintained, devoid of biotin treatment. Ultimately, total protein fractions were harvested and subjected to western blot analysis for the assessment of CLDN18.2 and S100A4. The inclusion of β-actin served to validate consistent total protein loading across all experimental groups. **e**-**f**. Analysis of data characteristics (**e**) and data distribution (**f**) was performed. **g**. Venn diagram showing the overlapped proteins and unique proteins associated with tumor tissue expressing CLDN18.2-TurboID. **h**. Heat map showing the top 20 up-regulated and down-regulated proteins. **i**. Radar chart showing the top up-regulated proteins. **j**. The histogram showing the relative abundance of S100A4, SERPINH1, Galectin-1, S100A10, and GPX4. **k**. S100A4 was silenced by siRNA in CAFs. **l**-**m**. The adhesion of GFP-labeled AGS cells upon co-culture with S100A4-knockdown CAF cells were assessed (**l**), and representative images are shown (**m**). The scale bar represents 200 μm. All data were shown as the mean ± SEM, and data were analyzed using One-way ANOVA test, **P < 0.01, ***P < 0.001 and ****P < 0.0001

**Fig. 7:**
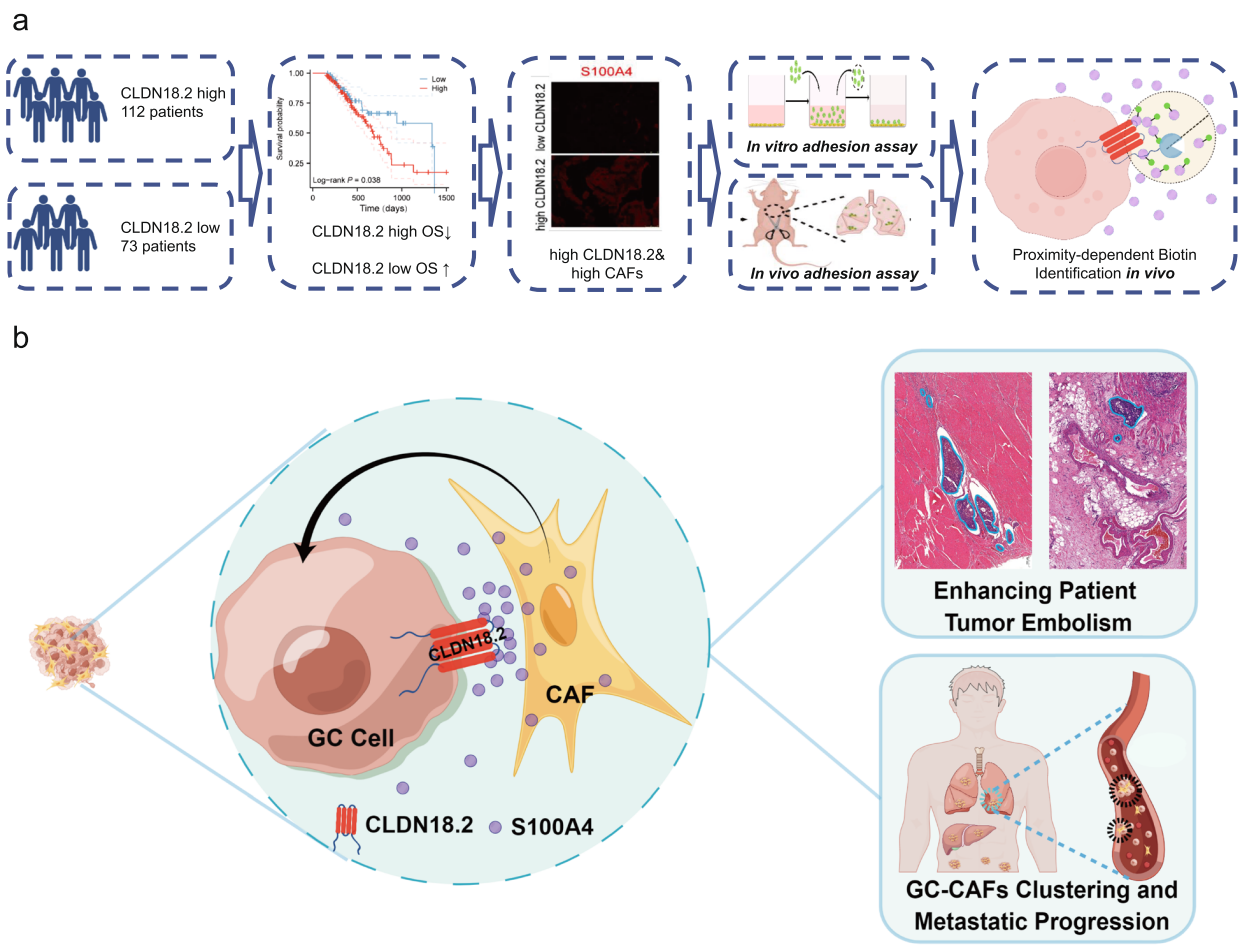
Workflow diagram and principle schematic. **a**. This schematic diagram presents the framework of this research, including collecting samples, labelling proteins, in vivo and in vitro experiments and protein-proximity labeling techniques. **b**. The rationale of regulatory mechanism of CLDN18.2 mediated juxtacrine interaction of GC Cells and CAFs

Since CLDN18.2 is an intercellular tight junction protein [4], the mechanism by which it mediates adhesion between CAFs and tumor cells deserves to be clarified. To address this question, we utilized proximity labeling assay to identify possible interacting proteins of CLDN18.2, including secreted and membrane proteins of CAFs (Fig. 6a). Initially, we fused and expressed biotin ligase TurboID in the extracellular segment of CLDN18.2 and PDGFRB, respectively, with the latter serving as a control plasmid (Fig. 6b). Next, we generated AGS cell line that stably expressed SP-TurboID-TM and TurboID-CLDN18.2 via lentiviral transduction. We then established a subcutaneous tumorigenesis model in nude mice, followed by in vivo proximity labeling by intraperitoneal injection of biotin into mice. Subsequently, we enriched biotinylated proteins using streptavidin beads and identified these proteins through liquid chromatography-tandem mass spectrometry (LC-MS/MS) analysis (Fig. 6c). Subsequently, we amalgamated western blot with proximity labeling assay to establish the interplay between CLDN18.2 and S100A4. The western blot investigations demonstrated a tangible interaction between CLDN18.2 and S100A4 exclusively within AGS cells co-cultivated with CAFs, whereas no such interaction manifested in AGS cells devoid of this coculture milieu (Fig. 6d). After conducting a relative quantitative analysis of mass spectrometry data, we found that the results of our principal component analysis (PCA) indicated a more significant difference between the experimental group and the control group than within each group, as indicated by the mountain plot (Fig. 6e, f). Subsequent differential protein analysis revealed that the control group, SP-TurboID-TM, had 444 co-captured proteins between the two repeat groups, whereas the experimental group, TurboID-CLDN18.2, had 460 co-captured proteins (Fig. 6g). To further understand the differences observed, we selected the top 20 up-regulated and down-regulated proteins for a heat map statistical analysis (Fig. 6h). Our results showed significant enrichment of secreted and membrane proteins in the TurboID-CLDN18.2 group, with S100A4, SERPINH1, Galectin-1, S100A10, and GPX4 exhibiting the most notable differences (Fig. 6i). Importantly, S100A4 is a crucial marker of CAFs and was significantly enriched in the TurboID-CLDN18.2 group (Fig. 6j). Furthermore, we employed siRNA-mediated suppression to effectively attenuate the expression of S100A4 in CAFs, thus assessing its implications for the migratory dynamics of gastric cancer cells (Fig. 6k). Transwell assays performed subsequently unveiled a discernible reduction in the migratory capacity of GFP-labeled AGS cells upon co-culture with S100A4-suppressed CAFs cells (Fig. 6l-m). These results illustrate that downregulation of S100A4 expression in CAFs cells potently impedes the migration of gastric cancer cells. Overall, our findings suggest that S100A4 interacts with CLDN18.2 or with the complexes formed by CLDN18.2 in the cell membrane. Moreover, the members of these protein complexes may be membrane receptor proteins. Finally, a validation on the role of S100A4 in the progression of CRC metastasis was constructed (Fig. 7).

## Discussion

CLDN18.2 is a tight junction protein primarily expressed in the normal gastric epithelium, but exposed during inflammation or cancer [24]. CLDN18.2 displayed a high positive expression rate in gastric cancer, while its expression is significantly increased in diffuse GCs compared to other GCs grouped by Lauren subtype [4]. Drawing much attention, CLDN18.2 has been recognized as a powerful therapeutic target for the development of monoclonal antibodies, bispecific antibodies, antibody-drug conjugates and cell therapies against gastric cancer [5, 27, 28]. Paradoxically, contrasting with the promising results of ongoing clinical trials as well as our clinical observations in this study that elevated CLDN18.2 expression is significantly associated with poor prognosis in gastric cancer patients, several studies have indicated that overexpression of CLDN18.2 inhibits gastric cancer cell metastasis [29]. The functional complexity about CLDN18.2 suggested its role in gastric cancer is deeply intertwined with the tumor microenvironment. In this study, we demonstrate that CLDN18.2-high gastric cancer cells exhibit enhanced adhesion to CAFs in the tumor microenvironment, thereby promoting gastric cancer metastasis and progression.

CAFs are among the most abundant and active stromal cells within the tumor microenvironment, playing crucial roles in modulating tumor malignancy and metastasis [30–32]. CAFs exert their effects on tumor progression through both paracrine signaling, in which soluble factors are secreted, and juxtacrine signaling, which occurs via direct cell-cell contact. While the former has been extensively studied in gastric cancer, with CAFs-secreted paracrine factors such as Lumican [33], IL-8 [34], WNT5A [35], extracellular vesicles [36], and others promoting tumor progression, the contribution of CAFs-gastric cancer cell juxtacrine signaling to gastric cancer progression remains unclear. Here we report for the first time that the tight junction protein CLDN18.2 plays a bridging role in mediating the juxtacrine interaction between gastric cancer cells and CAFs, and that this interaction contributes to gastric cancer metastasis and progression. Thus, it can be concluded that in the case of in vitro cancer cell lines & CDX models, CLDN18.2 exerted its function as a tight junction protein that slows down metastasis & progression, while in the presence of CAFs, CLDN18.2 promotes cancer cell-CAFs crosstalk and plays a malignant role in a CAF dependent manner.

We used proximity labeling technology [37] to elucidate CLDN18.2’s mechanism in promoting the juxtacrine interaction between gastric cancer cells and CAFs. We overexpressed CLDN18.2 in gastric cancer cells with an overexpressed biotin tag, and after subcutaneous injection into a mouse tumor model, we used mass spectrometry to confirmed that CLDN18.2 may produce a series of tumor-promoting effects by interacting with S100A4 in the tumor microenvironment. S100A4 serves as one of the most important markers of CAFs [38–41], existing literature reports suggest that S100A4 activates the TGF-β pathway, promoting the epithelial-mesenchymal transition of gastric cancer cells, and ultimately contributing to distant metastasis [42]. Additionally, S100A4 is an angiogenic factor that belongs to the calcium-binding protein family, and plays a critical role in the initiation and progression of cancer [43], whose upregulation is associated with a high incidence of tumor metastasis and poor prognosis [44]. However, the mechanism underlying the binding of CLDN18.2 to S100A4 in the tumor microenvironment remains elusive and requires further investigation.

In addition to driving cancer progression & metastasis through stimulating the malignant secretome of CAFs, the interaction between CLDN18.2 and S100A4 also directly drives cancer cell-fibroblast adhesion, which facilitates the physical retention of migrating cancer cells in fibroblast-rich stroma tissue or helps to cluster cancer cells and fibroblasts into large embolus. As shown by our in vivo evidences, the formation of cancer cell-CAF embolus in CLDN18.2 positive group was acute enough to suffocate mice to death in a short time. In the case of clinical practice, CLDN18.2 positive patients displayed higher burden of cancerous embolus, increasing the risk of metastasis and death from embolism. The disclosure of several clinical studies focusing on CLDN18.2-tageted therapies have took a giant leap towards the effective treatments against CLDN18.2-expressing tumors. However, a significant portion of patients still fail to experience therapeutic benefits from CLDN18.2 targeted therapies, emphasizing the necessity to improve therapeutic efficacy and to develop alternative therapies for patients failed to achieve long term response. By presenting the existence and functions of the CLDN18.2-mediated interaction between cancer cells and CAFs, our work shed light upon the potential involvement of CAFs in impeding CLDN18.2-targeted therapies. Even though we successfully block CLDN18.2, the effects present between tumor cells and CAFs, particularly the promotion of gastric cancer metastasis by the secretion of S100A4, remain intact. Since targeting CAFs has been recognized as a potential therapeutic strategy of cancer treatment [45], our research suggested that concurrently administering CLDN18.2-targeted therapies along with S100A4 inhibitors (or other CAF-targeted regimens) may disrupt this interplay and significantly enhance the therapeutic outcome. This approach holds significant promise for patients who have shown poor responsiveness to single-agent therapies.

### Supplementary Information

**Additional file 1.**

**Additional file 2.**

## Data Availability

All data presented and analyzed during this study are included in this article。.

## Abbreviations

CLDN18.2: Claudin-18.2
CAFs: Cancer-associated fibroblasts
S100A4: S100 calcium binding protein A4
GC: Gastric cancer
EMT: Epithelial-mesenchymal transition
PVDF: Polyvinylidene difluoride
H-score: Histochemistry score
ECOG: Eastern Cooperative Oncology Group
IHC: Immunohistochemistry
PCA: Principal Component Analysis
NAFs: Normal associated fibroblasts
OS: Overall survival
HR: Hazard ratio
FFPE: Formalin-fixed, paraffin-embedded
ANOVA: Analysis of variance
RT-qPCR: Quantitative real-time PCR
FBS: Fetal bovine serum
GO: Gene Ontology
KEGG: Kyoto Encyclopedia of Genes and Genomes
