# Supplementary material for: Claudin-18.2 mediated interaction of gastric Cancer cells and Cancer-associated fibroblasts drives tumor progression
Source: Cell Commun Signal. 2024 Jan 10;22:27. doi: 10.1186/s12964-023-01406-8 (PMC10777637; doi:10.1186/s12964-023-01406-8)
Supplement: Supplementary file 2 — Additional file 1. [file 12964_2023_1406_MOESM1_ESM.docx]

**Supplementary material for “CLDN18.2 Mediated Interaction of Gastric Cancer Cells and Cancer-Associated Fibroblasts Drives Tumor Progression”**

Shengde Liu ^1*^, Zizhen Zhang ^1*^, Lei Jiang ^1^, Miao Zhang^1^, Cheng Zhang ^1#^ and Lin Shen ^1#^

^1^ Key laboratory of Carcinogenesis and Translational Research (Ministry of Education/Beijing), Department of Gastrointestinal Oncology, Peking University Cancer Hospital & Institute, Beijing 100142, China.

*Shengde Liu and Zizhen Zhang contributed equally.

# Corresponding Authors:

Lin Shen, Key laboratory of Carcinogenesis and Translational Research (Ministry of Education/Beijing), Department of Gastrointestinal Oncology, Peking University Cancer Hospital & Institute, Beijing 100142, China. Department of Gastrointestinal Oncology, Key Laboratory of Carcinogenesis and Translational Research (Ministry of Education), Peking University Cancer Hospital & Institute, Beijing, China. shenlin@bjmu.edu.cn.

Cheng Zhang, Key laboratory of Carcinogenesis and Translational Research (Ministry of Education/Beijing), Department of Gastrointestinal Oncology, Peking University Cancer Hospital & Institute, Beijing 100142, China. Department of Gastrointestinal Oncology, Key Laboratory of Carcinogenesis and Translational Research (Ministry of Education), Peking University Cancer Hospital & Institute, Beijing, China. qenya_z@bjmu.edu.cn.

**Fig. S1**


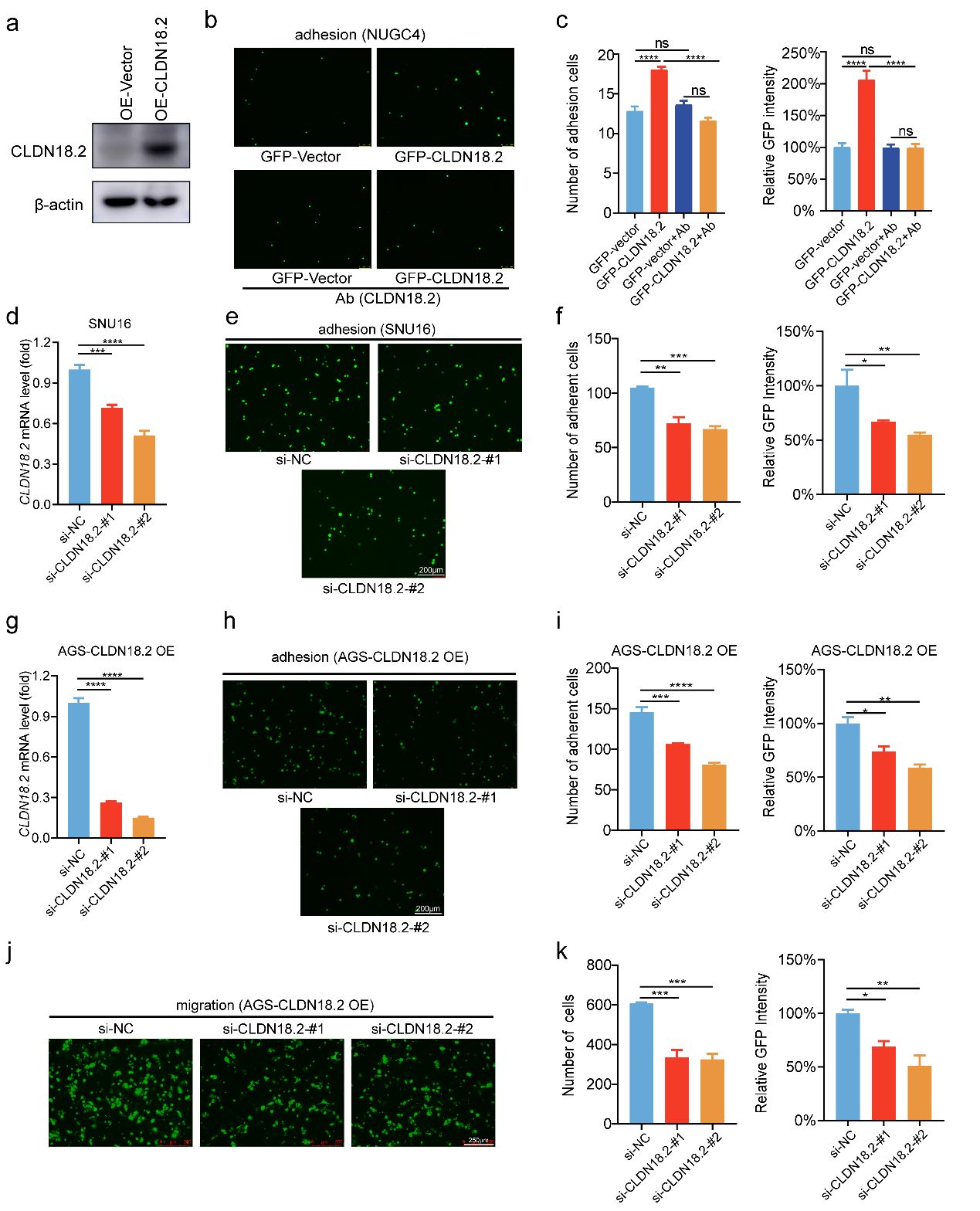


**Fig. S1**. a. Western blot showing expression levels of CLDN18.2 in the NUGC4 cells. b-c. Adhesion of NUGC4 cells expressing GFP or GFP-CLDN18.2 to CAFs was evaluated in the presence or absence of CLDN18.2 antibody blocking (b), and representative images are shown (c). The scale bar represents 100 μm. d. CLDN18.2 was silenced by siRNA in SNU16 cells. e-f. The adhesion between SNU16 cells with CLDN18.2 knockdown via siRNA and CAFs were assessed (e), and representative images are shown (f). The scale bar represents 200 μm. g. CLDN18.2 was silenced by siRNA in AGS expressing GFP-CLDN18.2. h-k. The adhesion and migration between AGS cells with CLDN18.2 knockdown via siRNA and CAFs were assessed (h-i), and representative images are shown (j-k). The scale bar represents 100 μm. All data were shown as the mean ± SEM, and data were analyzed using One-way ANOVA test, **P* <0.01, ***P* <0.01, ****P* < 0.001 and *****P* < 0.0001.

**Fig. S2**


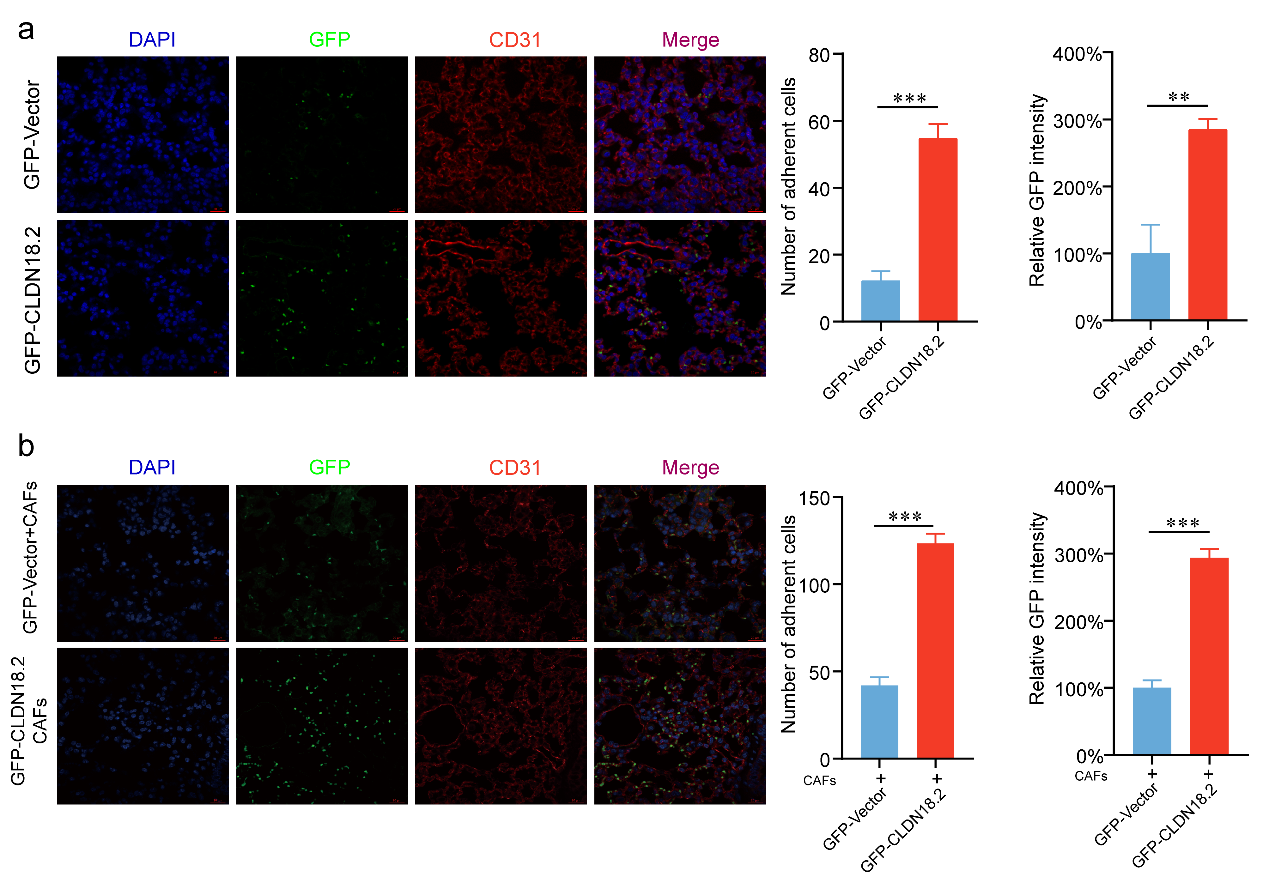


**Fig. S2**. a. GFP or GFP-CLDN18.2-expressing NUGC4 cells were injected into the mice tail vein, and the colonized cancer cells in the lungs were quantified by paraffin immunofluorescence assay after 24 hours. The blood vessels were stained with CD31 (red), and the nuclei were stained with DAPI (blue). Scale bar represents 40 µm. b. Mice were intravenously injected with AGS cells expressing either GFP or GFP-CLDN18.2, in combination with CAFs. The colonized cancer cells in the lungs were quantified by paraffin immunofluorescence assay after 24 hours. The blood vessels were stained with CD31 (red), and the nuclei were stained with DAPI (blue). Scale bar represents 40 µm. All data were shown as the mean ± SEM and analyzed using Student’s t-tests, ***P* < 0.01 and ****P* < 0.001.

**Fig. S3**


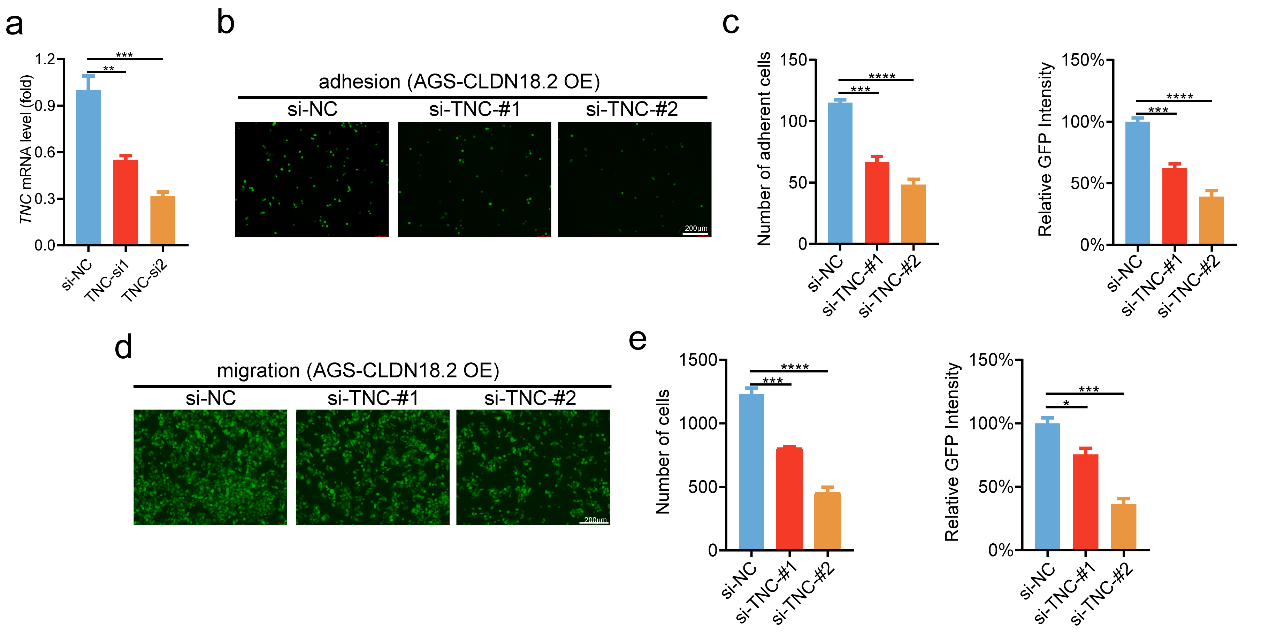


**Fig. S3**. a. TNC was silenced by siRNA in AGS expressing GFP-CLDN18.2. b-e. The adhesion and migration between AGS cells with TNC knockdown via siRNA and CAFs were assessed (b, d), and representative images are shown (c, e). The scale bar represents 200 μm. All data were shown as the mean ± SEM, and data were analyzed using One-way ANOVA test, **P* <0.01, ***P* <0.01, ****P* < 0.001 and *****P* < 0.0001.

**Table. S1: The clinical, pathological of CLDN18.2 positive**

**or negative gastric cancer patients**

| Characteristics | <40 | >40 | P value |
| --- | --- | --- | --- |
| n | 73 | 112 |  |
| Age, median (IQR) | 59 (52, 65) | 52.5 (38.75, 60) | < 0.001 |
| Gender, n (%) |  |  | 0.012 |
| male | 52 (28.1%) | 59 (31.9%) |  |
| female | 21 (11.4%) | 53 (28.6%) |  |
| ECOG PS, n (%) |  |  | 0.002 |
| 1 | 34 (18.4%) | 28 (15.1%) |  |
| 0 | 39 (21.1%) | 84 (45.4%) |  |
| Location, n (%) |  |  | 0.123 |
| GC | 61 (33%) | 102 (55.1%) |  |
| EGJ | 12 (6.5%) | 10 (5.4%) |  |
| Lauren classification, n (%) |  |  | 0.031 |
| Mixed type | 18 (9.7%) | 30 (16.2%) |  |
| Intestinal type | 29 (15.7%) | 25 (13.5%) |  |
| Diffused type | 26 (14.1%) | 57 (30.8%) |  |
| Differentiation, n (%) |  |  | < 0.001 |
| Moderate | 23 (12.4%) | 13 (7%) |  |
| Poor | 50 (27%) | 99 (53.5%) |  |
| T stage, n (%) |  |  | 0.009 |
| T1 | 0 (0%) | 1 (0.5%) |  |
| T2 | 6 (3.2%) | 2 (1.1%) |  |
| T3 | 27 (14.6%) | 24 (13%) |  |
| T4 | 40 (21.6%) | 85 (45.9%) |  |
| N stage, n (%) |  |  | 0.017 |
| N0 | 5 (2.7%) | 6 (3.2%) |  |
| N1 | 14 (7.6%) | 8 (4.3%) |  |
| N2 | 27 (14.6%) | 33 (17.8%) |  |
| N3 | 27 (14.6%) | 65 (35.1%) |  |
| M stage, n (%) |  |  | 0.010 |
| M0 | 30 (16.2%) | 26 (14.1%) |  |
| M1 | 43 (23.2%) | 86 (46.5%) |  |
| Pathologic stage, n (%) |  |  | 0.007 |
| Stage I | 4 (2.2%) | 1 (0.5%) |  |
| Stage II | 7 (3.8%) | 2 (1.1%) |  |
| Stage III | 20 (10.8%) | 23 (12.4%) |  |
| Stage IV | 42 (22.7%) | 86 (46.5%) |  |
| HER2 expression, n (%) |  |  | 0.098 |
| Negative | 38 (20.5%) | 72 (38.9%) |  |
| Positive | 35 (18.9%) | 40 (21.6%) |  |

**Table. S2: The clinical, pathological of CLDN18.2**

**positive or negative GC PDXs**

| Characteristics | high | low |
| --- | --- | --- |
| n | 5 | 5 |
| Age, mean ± SD | 65.8 ± 5.933 | 55.2 ± 17.57 |
| Gender, n (%) |  |  |
| male | 3 (30%) | 3 (30%) |
| female | 2 (20%) | 2 (20%) |
| ECOG PS, n (%) |  |  |
| 1 | 5 (50%) | 4 (40%) |
| 0 | 0 (0%) | 1 (10%) |
| Location, n (%) |  |  |
| GC | 5 (50%) | 3 (30%) |
| GEJ | 0 (0%) | 2 (20%) |
| Lauren classification, n (%) |  |  |
| Intestinal type | 4 (40%) | 4 (40%) |
| Diffused type | 1 (10%) | 0 (0%) |
| Mixed type | 0 (0%) | 1 (10%) |
| Differentiation, n (%) |  |  |
| Moderate | 3 (30%) | 4 (40%) |
| Poor | 2 (20%) | 1 (10%) |
| T.stage, n (%) |  |  |
| T3 | 0 (0%) | 2 (20%) |
| T4 | 5 (50%) | 3 (30%) |
| N.stage, n (%) |  |  |
| N0 | 0 (0%) | 1 (10%) |
| N1 | 0 (0%) | 1 (10%) |
| N2 | 3 (30%) | 2 (20%) |
| N3 | 2 (20%) | 1 (10%) |
| M.stage, n (%) |  |  |
| M0 | 0 (0%) | 2 (20%) |
| M1 | 5 (50%) | 3 (30%) |
| Pathologic.stage, n (%) |  |  |
| Stage III | 0 (0%) | 2 (20%) |
| Stage IV | 5 (50%) | 3 (30%) |

**Table. S3: Human primers for RT-qPCR**

| Primers name | Sequence (5’-3’) |
| --- | --- |
| *GAPDH*-F | CATGTACGTTGCTATCCAGGC |
| *GAPDH*-R | CTCCTTAATGTCACGCACGAT |
| *FAP*-F | CAAAGGCTGGAGCTAAGAATCC |
| *FAP*-R | ACTGCAAACATACTCGTTCATCA |
| *αSMA*-F | CTATGAGGGCTATGCCTTGCC |
| *αSMA*-R | GCTCAGCAGTAGTAACGAAGGA |
| *S100A4*-F | GATGAGCAACTTGGACAGCAA |
| *S100A4*-R | CTGGGCTGCTTATCTGGGAAG |

**Table. S4: siRNA sequence**

|  | Sequence (5’-3’) |
| --- | --- |
| CLDN18.2-siRNA-1 | GGUUUCACUGAUUGGGAUU |
| CLDN18.2-siRNA-2 | GCCUCCUGGUAUCCAUCUU |
| TNC-siRNA-1 | GCAUCUGUUUCGAAGGCUA |
| TNC-siRNA-2 | GCAGAACUCUCCUGUCCAA |
| S100A4-siRNA-1 | UGUCCACCUUCCACAAGUA |
| S100A4-siRNA-2 | GCAUCGCCAUGAUGUGUAA |
| Negative control | UUCUCCGAACGUGUCACGU |
