# Supplementary material for: Claudin-18.2 mediated interaction of gastric Cancer cells and Cancer-associated fibroblasts drives tumor progression
Source: Cell Commun Signal. 2024 Jan 10;22:27. doi: 10.1186/s12964-023-01406-8 (PMC10777637; doi:10.1186/s12964-023-01406-8)

Unedited blot and gel images

Full unedited blots for Figure 3B

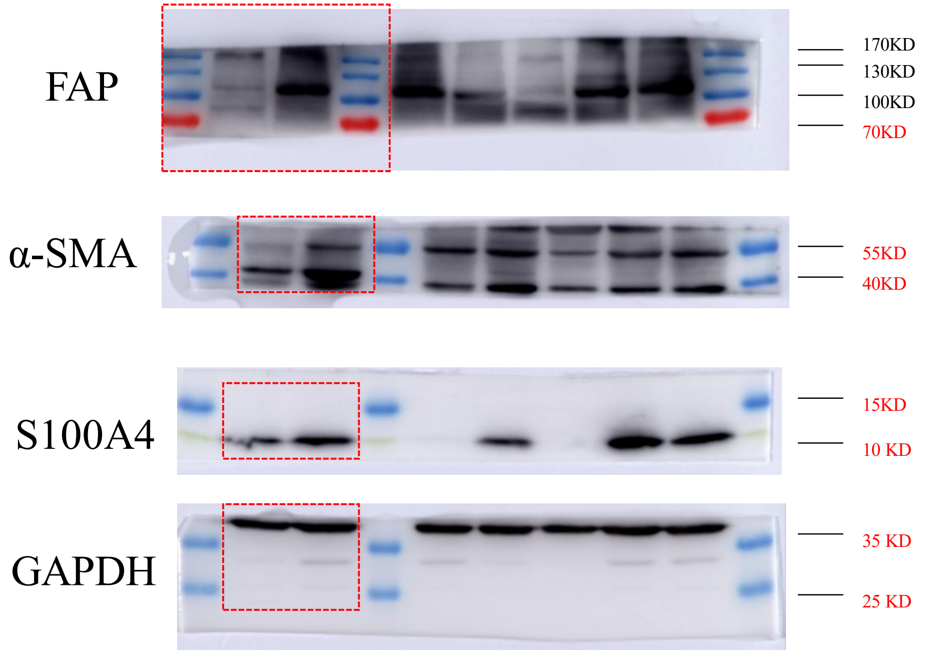

Full unedited blots for Figure 3C

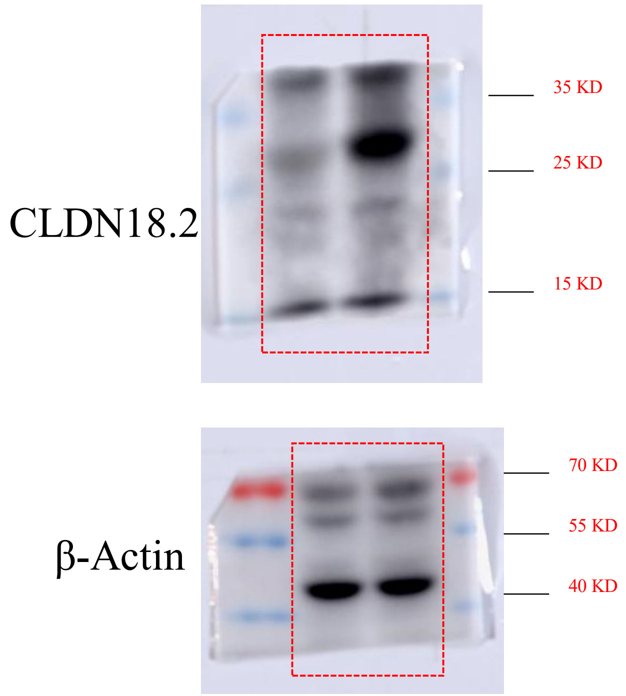

Full unedited blots for Figure 6C

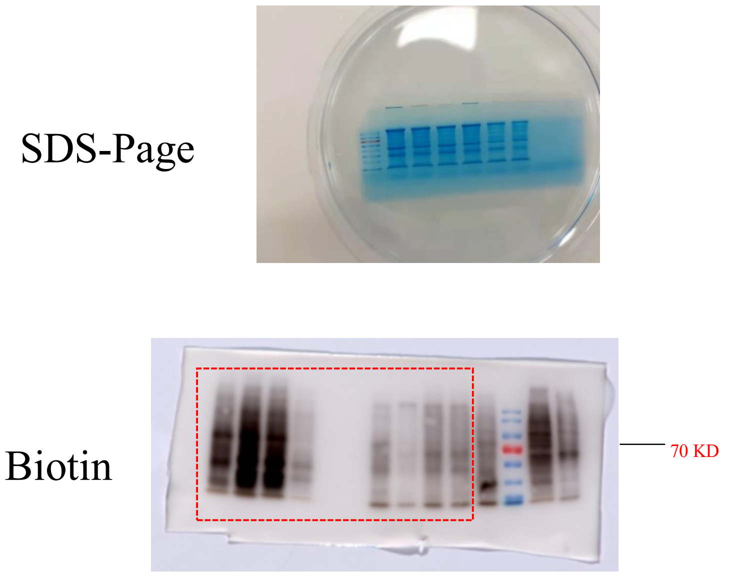

Supplement: Supplementary file 3 — Additional file 2. [file 12964_2023_1406_MOESM2_ESM.pdf]
